# A Comprehensive Review of Methods and Equipment for Aiding Automatic Glaucoma Tracking

**DOI:** 10.3390/diagnostics12040935

**Published:** 2022-04-08

**Authors:** José Camara, Alexandre Neto, Ivan Miguel Pires, María Vanessa Villasana, Eftim Zdravevski, António Cunha

**Affiliations:** 1 Departamento de Ciências e Tecnologia, Universidade Aberta, 1250-100 Lisboa, Portugal; jcrcamara@hotmail.com; 2 Instituto de Engenharia de Sistemas e Computadores, Tecnologia e Ciência, 3200-465 Porto, Portugal; alexandre.hc.neto24@gmail.com; 3 Escola de Ciências e Tecnologia, University of Trás-os-Montes e Alto Douro, Quinta de Prados, 5001-801 Vila Real, Portugal; impires@it.ubi.pt; 4 Instituto de Telecomunicações, Universidade da Beira Interior, 6200-001 Covilhã, Portugal; 5 Centro Hospitalar Universitário Cova da Beira, 6200-251 Covilhã, Portugal; ma.vanessa.villasana@chcbeira.min-saude.pt; 6 UICISA:E Research Centre, School of Health, Polytechnic Institute of Viseu, 3504-510 Viseu, Portugal; 7 Faculty of Computer Science and Engineering, University Ss Cyril and Methodius, 1000 Skopje, North Macedonia; eftim.zdravevski@finki.ukim.mk

**Keywords:** glaucoma, image processing, glaucomatous papilla, clinical data, disease tracking, eye

## Abstract

Glaucoma is a chronic optic neuropathy characterized by irreversible damage to the retinal nerve fiber layer (RNFL), resulting in changes in the visual field (VC). Glaucoma screening is performed through a complete ophthalmological examination, using images of the optic papilla obtained in vivo for the evaluation of glaucomatous characteristics, eye pressure, and visual field. Identifying the glaucomatous papilla is quite important, as optical papillary images are considered the gold standard for tracking. Therefore, this article presents a review of the diagnostic methods used to identify the glaucomatous papilla through technology over the last five years. Based on the analyzed works, the current state-of-the-art methods are identified, the current challenges are analyzed, and the shortcomings of these methods are investigated, especially from the point of view of automation and independence in performing these measurements. Finally, the topics for future work and the challenges that need to be solved are proposed.

## 1. Introduction

Glaucoma is an optic neuropathy disease associated with a characteristic reduction in the visual field leading to total loss of vision, affecting approximately 80 million people worldwide [1]. In Western countries, vision loss is frequently associated with open-angle glaucoma, whereas in Far East regions, is more common due to angle-closure glaucoma, showing the variations of glaucoma incidence between different racial and ethnic groups [2,3,4]. Different risk factors are responsible for this disease, including family tendency, marked myopia, and genetic factors that play an essential role in the inheritance of glaucoma [5]. The incidence in first-degree relatives seems to increase the risk of developing the disease, which usually progresses slowly and asymptomatically [6]. Central visual acuity is usually preserved until later stages of the disease, with progressive loss of the peripheral visual field related to the nerve fibers and consequently, leading to irreversible blindness [7]. In 2010, the estimated global prevalence of glaucoma was 60.5 million people, with 79.6 million cases expected in 2020 [8].

Didactically, the human eye can be divided into portions: ocular adnexa (i.e., eyelids and lacrimal glands), anterior segment (i.e., cornea, lens, iris, and pupil), posterior segment (i.e., vitreous, retina, macula, and optic papilla) [9]. The adnexal glands secrete the tears that maintain constant hydration on the ocular surface. The anterior segment structures make up a set of structures with dioptric power to focus light on the retina, such as the lacrimal system, sclera, cornea, iris, and lens. The anterior portion of the eye can be evaluated by biomicroscopy using a slit lamp that cuts light into the structures. The posterior segment visualization can be performed directly using ophthalmoscopes or by background biomicroscopy, or indirectly using retinal photographs and indirect ophthalmoscopy.

The assessment of the optic papilla is the most important diagnostic element in screening and for the control of disease progression [10]. However, eyes with media opacity, small pupils, and high ambient luminosity can affect the characterization of the papilla. In general, the ophthalmological consultation goes through the following steps:

- **Step 1:** the collection of the patient’s history and an ophthalmological examination, refractometry to verify the degree of axis, verification of eye movements, biomicroscopy for visualization from the anterior part of the eye, direct ophthalmoscopy for visualization of the fundus of the eye or fundus biomicroscopy, tonometry (measurement of eye pressure) and a presumptive diagnosis of pathologies that must include glaucoma screening;
- **Step 2:** the negative cases will have semiannual or annual returns, and the positive cases are forwarded for subsidiary examinations and treated. Patients undergoing treatment are monitored and may require treatment adjustments according to their evolution.

Glaucoma screening is mandatory in eye care [11]. Glaucoma is confirmed by the presence of characteristics that can be directly observed in the optic papilla, such as sectorial or diffuse excavation enlargement due to the death of ganglion cells that extend from the retina to the optic nerve, and these observations are correlated with data from the anamnesis [12]. Screening is highly dependent on the examiner’s professional experience. It relies on subjective data to quantify the damage resulting from the loss of nerve fibers, usually using a ratio between the limits of the papilla and the excavation [13]. Many cases of false negatives are dispensed with, even on the first visit [14,15].

The evolution of glaucoma is related to risk factors, such as increased intraocular pressure, advancing age, the presence of cataracts, myopia, race, and family history [16]. The progression of the disease leads to the progressive loss of retinal ganglion cells in the peripapillary area and the increase in neuroretinal rim thinning, usually asymmetrically between the eyes. Structural glaucomatous changes occur before the functional detection of glaucoma defects in the visual field. On the other hand, the Ocular Hypertension Treatment Study (OHTS) that followed eyes with ocular hypertension through photographs revealed that a significant number of patients had visual field alterations even before the detection of the defect in the neural rhyme [17], leading to false-negative results in cases where the tracking is based only on observation of the characteristics of the glaucomatous papilla.

The treatment of glaucoma is based on controlling the progression of the disease through drugs that control ocular pressure by reducing the production of aqueous humor and resistance to the elimination of intraocular fluids. In addition, filtering surgeries are used in certain situations such as failure of clinical treatment and unfavorable social situations, among others.

In most cases, glaucoma is asymptomatic, and screening for the disease must be performed in the routine ophthalmological examination, based on three main points: the characteristics of the optic papilla, the intraocular pressure, and the visual field [18]. The increased ratio between the vertical diameter of the cup and the papilla is greater than 0.6 [19]. Elevated intraocular pressure (IOP) is one of the main risk factors for vision loss resulting from open-angle and closed-angle glaucoma, and it is the only factor subject to modification [20]. Epidemiological studies and clinical trials have indicated that optimal control of IOP helps reduce the risk of damage to the optic nerve and slow the progression of the disease. Decreasing IOP is the only intervention ever demonstrated to prevent vision loss resulting from glaucoma. Cases without a visible increase in optical cupping, but with increased eye pressure, should be monitored. Visual field testing identifies, locates, and quantifies the degree of visual field loss [21]. The existence of visual field damage may indicate the presence of a medium to advanced level of disease. Therefore, it is essential to monitor the visual field to determine the instability of the disease, as shown below. The use of automatic diagnostic support systems provides more reliable diagnostic results, with less variability among observers, as well as optimizing and allows for earlier treatment initiation with better visual benefits [22,23]. These systems can automatically analyze and interpret photographic images of the optical papilla and data from the anamnesis, constituting an important tool for tracking and diagnosing glaucoma. The automatic tracking of the glaucomatous papilla presents good results when analyzing papillary image databases, sampling images in vivo, and comparing characteristics of the papilla image, family history, and age [24]. However, a small number of samples were used with little pathological variability, making them unsuitable for practical use. Its acceptance by experts depends on the proof of reproducibility using real data.

This paper presents a review of the literature published since 2015 dealing with the main methods that can contribute to automatic glaucoma screening, highlighting the main contributions and limitations. In addition, it presents the main available tracking systems and identified the main scientific challenges in this area.

The article is organized as follows. In Section 2, the fundamentals for screening for glaucoma are presented. Next, Section 3 presents the image acquisition equipment currently available for screening glaucoma, along with their characteristics. In addition, Section 4 presents the main systems available for automatic tracking of glaucoma, highlighting the features provided. Finally, the main challenges for glaucoma screening are presented in Section 5, with the main conclusions summarized in Section 6.

## 2. Fundamentals of Glaucoma Screening

**Figure 1:**
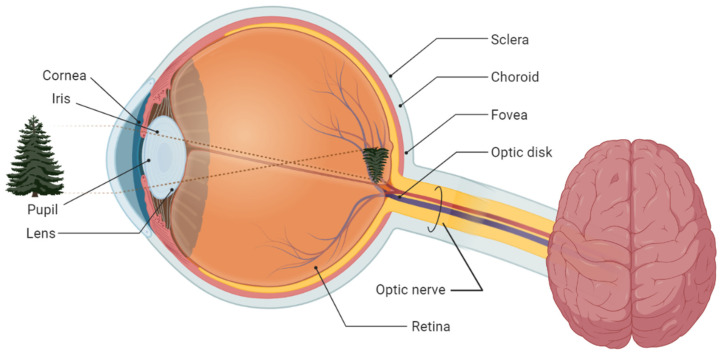
Scheme of the human visual system.

The human eye captures the light from the surrounding environment and sends it through the optic nerve to the brain, as shown in Figure 1.

**Figure 2:**
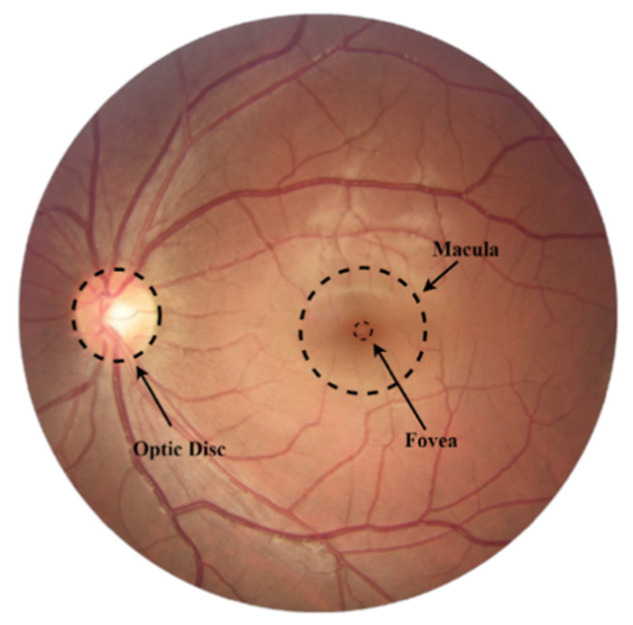
Human retina.

The external light passes through the cornea and is redirected using the lens to focus the image on the retina [25]. The retina converts light into electrical stimulation transmitted by the optic nerve to the occipital cortex, where the images are interpreted. By opening or closing, the iris has the function of controlling the amount of light that enters the posterior part of the eye and reaches the retina [26]. The retina can be photographed as shown in Figure 2.

**Figure 3:**
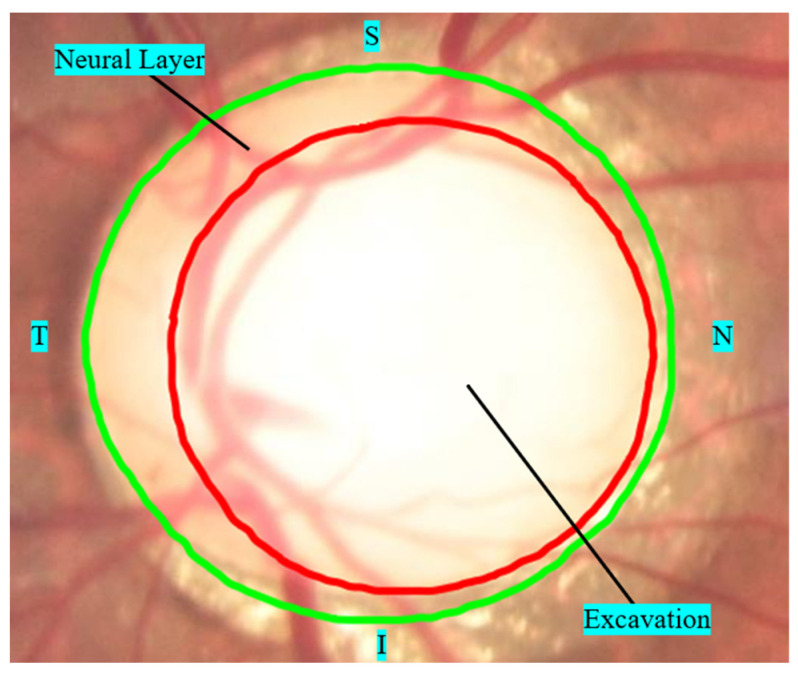
Optical papilla zone.

The macula region has a greater concentration of cones (responsible for converting the light into electrical stimulation), which allows for viewing this region in greater detail. These cells connect to ganglion cell axons (which make up the retinal fibers) with a characteristic arcuate distribution that converges on the optic disc [27]. In the optic disc, these fibers form the neural layer, which has an orange coloration different from the retinal tissue and the cup in the center of the papilla, and it is also lighter (see Figure 3) [28].

This retinal image analysis can provide information about pathological changes caused by eye diseases, along with signs of systemic diseases such as hypertension, arteriosclerosis, diabetes mellitus, and glaucoma [27,29]. Glaucoma is a disease that damages the optic nerve, with consequent loss of vision from the periphery field to the center until total blindness is incurred [20]. This disease appears due to the death of fibers, manifesting itself with the change in the characteristics and limits of the neural layer of the papilla (glaucomatous papilla), which is marked by the appearance of a defect in the neural layer (notch) resulting from sectorial atrophy [27]. As an asymptomatic disease, patients do not seek an ophthalmological consultation in time, in most cases, leading to partial or total loss of vision. For this reason, population mass-screening is essential for its early diagnosis and treatment [30,31].

### 2.1. Glaucoma Tracking

Glaucoma screening should be performed routinely by an ophthalmologist and is based on data from anamnesis, observation of the fundus of the eye, and intraocular pressure values; when available, visual field examination is also used. The ophthalmological history provides clinical data of diagnostic importance to the various ocular pathologies, including glaucoma screening, with data related to an external inspection of the eyes, recording of complaints and duration of visual symptoms, personal data and family history, and data from the eye exam [32,33]. These data are summarized in Table 1.

The observation of the fundus of the eye allows for the detection of characteristic changes in the optic papilla caused by glaucoma, which is why it is essential for tracking glaucoma [34]. Visualization of the optic papilla can be performed directly, e.g., through the direct ophthalmoscope, or indirectly, through indirect ophthalmoscopy and retinal photographs that are taken with photographic cameras. The photographic documentation is of great value, as it allows quantitative measurements of the external edge, the internal excavation, and the vertical axis of the right and left papillae.

Another method of detecting this disease is by measuring eye pressure, since it is a risk factor associated with glaucoma presence, and it can be measured using a device called a tonometer. When this intraocular pressure (IOP) is as high as 21 mmHg, it can be defined as ocular hypertension. A clinical study called the Ocular Hypertension Treatment Study [35] included 1636 patients with IOP above 24 mmHg that were randomized and treated to decrease 20% of the IOP. After five years, only 9.5% of untreated people progressed to glaucoma, and 4.4% of those treated developed glaucoma, demonstrating that IOP alone is not a characterization factor for inclusion in the glaucoma group. Other parameters should be considered to provide a more sensitive outcome and greater chances of avoiding false-negative or false-positive results [30,36,37].

**Figure 4:**
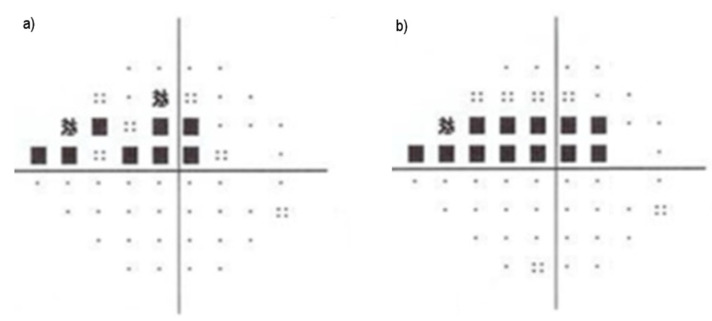
Visual field diagrams. (**a**) shows the initial field of view; (**b**) shows the posterior field of view, illustrating the worsening of glaucoma. The lightest dots represent islands of vision, and the squares with a gradation of gray (partial visual loss) to black (complete loss of vision) that increase in number as the disease progresses.

The visual field, for example, is important for the identification, localization, and quantification of the degree of visual field loss in regions with an atrophy of the ocular fibers. The field of vision showing an initial lesion can be seen in the diagram in Figure 4a,b, which shows the aggravation of glaucoma. It is obtained with a perimeter that is not always available in initial consultations where glaucoma tracking is performed.

If the visual field is normal, the patient may be submitted to other strategies that will allow the identification of early glaucoma through additional structural tests such as confocal laser ophthalmoscopy, optical coherence tomography (OCT), and laser polarimetry (GDx) [38]. Functional tests designed to detect fiber layer changes before visual field changes occur include frequency doubling perimetry (FDP) and short-wavelength automated perimetry, but their role in clinical practice has diminished over the years [39]. These tests have limitations, such as the inability to evaluate eyes with high myopia in large optic discs, or the presence of large areas of peripapillary atrophy. In these cases, the patient must be assessed as a whole, and there must be a correlation of eye pressure, structural, and functional data. Additionally, the characteristics of the papilla can be observed qualitatively by the examiner’s observation, or quantitatively, by measuring the structures of the papilla [40]. These characteristics can be seen through the texture, the ratio of the measurement of the outer and inner edges of the optic papillary region, the size of the cup around the contralateral papilla, defects located in the neural layer (notch), sectorial layer atrophy of nerve fibers, increased mono- or binocular cupping, hemorrhage in the neural layer of the papilla, increased vertical diameter, and diffuse thinning of the neural ring [41]. However, there will only be diagnostic certainty with the progression of the disease, that is, with an increase in cupping corresponding to an alteration in the visual field.

**Figure 5:**
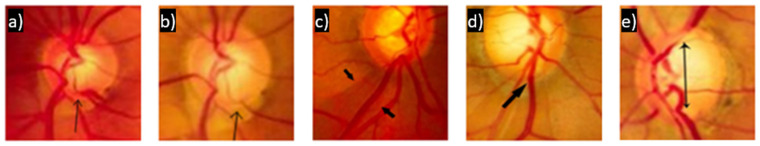
Example of changes in the glaucomatous papilla. (**a**,**b**) show the loss of nerve fibers in the inferior pole of the optic papilla; (**c**) the appearance of a wedge-shaped defect (localized RNFL defect–Hoyt sign); (**d**) shows the atrophy located in the inferior pole; (**e**) shows the increase in the vertical diameter of the papilla.

Specialists usually observe the delineation of the neural layer and the excavation by color nuances, or the morphology of vascular emergence. Sectoral atrophy of the fiber layer can cause a “collapse” of the vessel, giving a distorted appearance of the vascular emergence that helps delimit the internal cavity. Figure 5 exemplifies some characteristics of the glaucomatous papilla.

Figure 5a,b show the progression of an area located in the inferior pole of the neural layer evidenced by the deflection of a vessel at 5:00 a.m. Figure 5c shows an increased excavation of 50 to 60% and an area of peripapillary sectorial atrophy from 7:00 to 8:00. Figure 5d shows an enlarged cup reaching about 70% of the neural layer and area of atrophy at 6:00, where the emerging vessel defenses. Figure 5e shows the excavation of about 70 to 80%, with an increase in the vertical axis, whose lower delimitation is marked by the presence of vessel deflection at 6:00. The thickness of the neural layer varies according to the number of fibers, being thicker in the inferior pole (I), followed by the superior pole (S), nasal (N), and temporal (T) poles, a fact that constitutes the ISNT rule. Changing the ISNT rule can characterize glaucomatous papillae. Atrophy of the inferior and superior poles of the papilla increases the vertical axis of the cup [42]. Moreover, a diffuse increase in the cup, usually asymmetric between the two eyes, can also be observed.

Despite the development of new technologies that quantitatively measure the topography of the optic papilla and the thickness of the retinal nerve fiber layer, photography remains the gold standard for structural analysis. It should never be underestimated [43]. However, there is no absolute consensus on the criteria used to define the glaucomatous papilla. Therefore, quantitative measures of cupping are assumed. The papilla is an oval, orange structure with a diameter ranging from 1 to 3 mm. The relationship between the inner edge of the cup and the outer limit of the papilla varies from 0.1 to 1.0. Cupping of 0.2 to 0.3 is considered physiological. The closer the cup approaches 1.0, the closer to the disease is to showing an advanced state. The following conditions were established to make the automatic detection of suspected glaucoma excavation more objective [10,44]:

▪ The vertical axis of the cup is greater than 65% of the vertical axis of the outer edge of the papilla in large papillae (>1.5 mm) and 50% of the vertical axis in small papillae (<1.5 mm).
▪ The limit of papilla cupping is less than 20% of the outer boundary of the papilla, including the localized area of neural layer atrophy (notch).
▪ Asymmetry of the internal excavation is greater than 20% between the optic papillae of the right and left eyes.
▪ Defects in the nerve fiber layer in the peripapillary region, associated or not with a suspicious thinning of the nerve fiber layer, as exemplified in Figure 5c, where an area of atrophy is visible in the path of the fibers from 7:00 and 8:00.

The ICO Guidelines for the Treatment of Glaucoma define other measures as diagnostic criteria, including a cup ≥ 0.5 (e.g., a 0.9 × 0.8 cup shows advanced glaucoma) [45]. The authors of [46] added clinical parameters in glaucoma screening (eye pressure, race, age), in addition to papillary measurements.

Khunger et al. [47] argue that automated classification methods detect glaucomatous papilla by their appearance. However, other clinical factors can make it challenging to track glaucomatous papillae related to pathologies that progress with media opacity, such as dystrophies and degenerations that can develop with corneal opacities, cataracts, inflammatory processes of the ocular adnexa, conjunctiva, sclera, anterior uveitis, iritis, and later uveitis, among others. In addition, high ametropias can create technical difficulties for obtaining the focus of the papilla, as well as eyes with small pupillary slits that can obliterate the limits of the papilla, impairing the visualization of the outer boundary of the papilla as well as the peripapillary area, abnormal changes in eye movement (strabismus, nystagmus), lack of patient cooperation, the anatomical variability of optic papilla insertion, atrophic papillae that may give the impression of increased cupping, papillary drusen, systemic and ocular pathologies with posterior pole involvement such as diabetes, hypertension, rheumatologic and neurological conditions that accompany cranial hypertension, size and insertion of the optic papilla, optic neuropathies, and media opacity can all affect the observation of the characteristics of the papilla.

In clinical practice, asymmetry of the papilla between the two eyes is considered suggestive of glaucoma, especially in the vertical axis, which, when present, is strongly suggestive of retinal neuron damage, even with normal ocular pressure.

### 2.2. Metrics Used for Glaucoma Assessment

The papilla is an oval structure in the nasal region of the retina, through which the fibers that bring electrical stimuli formed by the light that penetrates the eye by specialized retinal cells pass. It is a region susceptible to damage from increased eye pressure. The neural layer of the papilla, formed by axons of fibers from the retina, presents significant anatomical variability, with orange coloration, a slightly whiter coloration of the internal excavation, and an average diameter of 1 to 3 mm. The attenuation of colors between the retina and the papilla’s neural layer allows for the delimitation of the papilla’s external excavation and the papilla’s internal excavation of the nerve fiber layer. The inner boundary of the excavation is not uniform and has a highly variable shape. The papilla’s outer limit, the cup’s diameter, and the cup’s depth are greater. The papilla shows variability in the size and inclination of the insertion. The evaluation of the excavation may be impaired in many cases, including papillary edge edema, sectorial papillary atrophy, peripapillary atrophy, and papillary drusen. The size of the papilla is independent of age, gender, and weight. It is associated with ethnicity and the degree of the axis. In myopic eyes, the papilla is usually more prominent than in farsighted eyes. The relationship between the size of the cup and the outer limit of the papilla is used as an assessment method. Even in patients without glaucoma, the predominant relationship between the cup and the outer boundary of the papilla is less than 0.3 in most patients. Only 1 to 2% of normal individuals have cups above 0.7.

The automatic segmentation of the boundaries of the optic disc and optic cup is a process to help identify and assess the progression of the glaucomatous disease [48]. However, it is a complex process, considering that the subtlety and variability of the anatomy of the nerve fiber layer, irregular contours due to glaucomatous damage to the nerve fibers (notch), and the presence of blood vessels, in some cases, impair the visualization of the excavation limits. In other cases, these conditions can even indirectly aid visualization by the deflection that they suffer in the regions of atrophy of the fiber layer.

The optical papilla segmentation methods aim to quantify the measurements of the disc and optical cupping more precisely [49]. Manual or automated methods can perform segmentation, but these have their limitations, and low image resolution and anatomical noise are cited as the main limiting factors. In addition, fixing the exact limits between the normal and glaucomatous papillae becomes a constant challenge due to the local anatomical variability and the difficulty of establishing precise excavation boundaries.

The diagnosis of glaucoma can be made using assessment metrics in fundus photographs through measurements of the optical axis limit, cupping, neural portion area, ISNT, vertical axis, and notch [50]. Among the different classification methods proposed by several authors for the diagnosis of glaucoma, most were based on the extraction of anatomical features, and a small number were based on the use of image textures [49,51,52,53,54,55]. The combination of these methods with clinical data has the potential to become an effective methodology for screening glaucoma.

Thakkar et al. [56] tested 150 private images from an ophthalmological hospital, analyzing the region of optic nerve atrophy through characteristic changes, such as the presence of ISNT and the notch calculated through circular bridges, and proposed their classification into groups: with glaucoma, without glaucoma, and unresolved cases. The methodology is divided into the image pre-processing, cropping and fitting, image noise removal, pixel detection, size coloration, transformation, dilation, the performance of segmentation of NO, disc, and blood vessels, the calculation of the boundaries and the area of the neural layer, the analysis of the intersection of disc and vessels, the measure of cupping, and the second phase results in normal, glaucoma, and unresolved groups. It does not report results or control groups used.

In [57], Thakkar et al., proposed an algorithm that allows the assessment of various eye diseases based on the location and segmentation of the optic disc and detection of peripapillary atrophy (useful in the early diagnosis of glaucoma). It uses the diversification of brightness intensity and colors to locate the papilla, removing the vascular structure in the optic disc region. The process, implemented in the MATLAB 2014 prototype and tested with images from the DRIVE base, presents images with different retinal diseases such as diabetic retinopathy and glaucoma, obtaining an accuracy of 82.14% and 75%, respectively, and an accuracy of 81.82% for the prediction of the area of peripapillary atrophy.

Miller et al. [58] proposed a comparison model for measuring cupping (CDR) on 422 fundus images obtained by portable non-mydriatic cameras and traditional mydriatic cameras that were remotely classified by experts using the CDR measurement > 0.7 and possible evidence of glaucoma, such as the presence of a notch. There were no differences between the CDR measurements in the photos taken by portable and traditional cameras. Thus, they concluded that mobile cameras could facilitate the remote evaluation of optical disc images and photography with mydriasis.

Viquez et al. [59] used Arclight, a low-cost right ophthalmoscope, to obtain background photographs attached to a smartphone and described the first low-quality photo fusion method compared to images from traditional cameras.

In [60], Wintergerst et al. evaluated optical cup measurements in photos taken on smartphones of papillae with dilated and non-dilated pupils, with 74% accuracy in eyes without dilation and 98% with dilation (which allowed the better quality of imaging). The optic papillary threshold was fully visualized in only 46% of non-dilated images vs. 94% of dilated images, and demonstrating that the evaluation of optic papilla is possible in non-dilated eyes, but it underestimates the risk for glaucoma.

Maccormick et al. [61] proposed a diagnostic method for glaucoma based on the spatial analysis of the excavation. They introduced a probabilistic spatial model that calculates an index of disc deformation by examining 3280 eyes of people aged 40 to 80 years, among whom 149 were diagnosed with glaucoma. The algorithm distinguished healthy and glaucomatous discs with 91.0% and 99.6% accuracy, respectively, requiring fewer samples than does deep learning. In addition, they performed an ORIGA and RIM-ONE-based analysis. In contrast to the strategies involved in machine learning, the spatial model allows for the evaluation of the deformation index and the relevant characteristics of the optical disc.

Claro et al. [62] presented a methodology for automatic optical disc detection based on the extraction of entropy and color characteristics in different models to classify retinal images. Segmentation brought good results, with accuracy higher than 83%, while the classical value in the literature was 70%.

In [63], Santos et al. presented an automatic method of detection and segmentation of the optical disc for medical retinal images. The results were performed using three databases: RIM-ONE, DRISHT-GS, and DRIONS-DB, with the extraction of the red channel and delimitation of the region of interest. The determination of the center of the optical disc was the region with the most significant color intensity, and the delimitation of the boundaries and the segmentation of the optic disc was carried out. An accuracy 83%, when evaluated with a required threshold of 70%, represents the traditional value found in the literature.

Joshi et al. [64] tested 138 optic disc images (33 normal and 105 with glaucoma) using geometric parameters of the optic disc, estimated disc, and cup calculation compared to the limits demarcated by three experts.

Maheshwari et al. [26] proposed an automatic papilla system with bit-plane cuts in red, green, and blue channels, in addition to binary local characteristics based on different pixels in the optical papilla region, with an accuracy of 99.30%, high sensitivity, and specificity capable of assisting experts in mass screening.

In general, the authors detailed different methods to locate the papilla and analyze characteristic elements that differentiate the normal and glaucomatous papillae in different evolutionary phases of glaucoma. Furthermore, they achieved good results by obtaining images through the use of portable cameras and smartphone cameras, allowing other specialists to transmit photos. Therefore, the automated analysis of pictures of the papillae of both eyes is associated with clinical data and visual field, with results closer to reality. However, because several authors’ results use only the automated analysis of papilla images, there is a need to analyze clinical data to classify normal or glaucomatous papillae and determine a clinically “suspicious” group with normal papillae.

The various forms of automated papilla analysis aim to establish a methodology capable of recognizing elements of the glaucomatous papilla. The different methods based on the extraction of other characteristics proved to be viable, with a qualitative level; however, the lack of a standardization of parameters allowing for the comparison of the results of an optical papilla using different methodologies makes it difficult to assess the diagnostic advantages presented. The use of 2D images obtained by smartphone, analyzed by direct ophthalmoscope, did not show inferior quality compared to stereoscopic images. Miller et al. [58] concluded that traditional and portable cameras could facilitate the remote assessment of disc images in other centers. However, Wintergerst et al. [60] concluded that the diagnosis of glaucomatous papilla could be underestimated in images without dilating the pupil. In fact, the limitation of pupil size can confirm that. One of the problems is that there is no single, universally accepted quantitative parameter for standard papilla measurements. In addition to the anatomical variability of the dimensions, the insertion of the papilla, other physiological elements, such as the presence of drusen, pits, and pathological factors, such as diffuse and sectoral atrophies, can result in structural variations that can lead to confusion in screening for glaucomatous papillae. The presence of blood vessels can interfere with the estimation of the excavation, or even help it. Because of the vascular deflection caused by decreased tissue support of the neural layer, as well as the segmentation processes, with vessel suppression, the diagnostic steps may not be helpful in cases of moderate to severe excavations.

The disadvantage of using smartphones to take photographs of the papilla is the physical limitation of pictures to capture small pupils, as well as the right pupil reflex activated by flashes, which may underestimate the risk for glaucoma. Given these elements, it can be concluded that, for the success of tracking glaucomatous papillae through papillary images, it is necessary to continuously increase the image database data to define a glaucomatous papilla based on its non-glaucomatous morphological diversities, and to develop technological improvement of the smartphone camera, enabling it to enlarge the image field and reduce the need for flash.

The diagnostic and therapeutic approach to glaucoma in resource-poor regions presents unique challenges. Lack of means to pay, low adherence to treatment, and lack of provider training and knowledge are obstacles to good therapy. In addition, the manifestation of the disease is asymptomatic, making most patients unaware of the condition. At the time of diagnosis, many people have already lost a large part of their vision. Other factors that make the diagnostic approach difficult are the long distances travelled to medical establishments and the insufficiency of equipment, internet connections, and professionals in the health sector. A diagnosis of open- or closed-angle glaucoma requires medical and surgical intervention to prevent vision loss and maintain quality of life.

## 3. Retinal Visualization Equipment

### 3.1. Retinographer

Retinography and digital angiography are images obtained using devices called retinographs [65,66]. They are intended to observe and record images of areas of the fundus of the eye, including the retina, choroid, optic papilla, and blood vessels. These images are obtained in a non-invasive and painless way that allows the diagnosis and documentation of the disease progression. The most modern retinographs are found in Brazil for prices between USD $50,000 and USD $100,000, with prices soaring even higher with the inclusion of optional features [65]. In general, equipment on the market uses a color image sensor and often, one or two monochrome sensors, for taking photographs after injecting intravenous contrast. The use of two types of detectors in the same equipment results from a limitation existing in the use of color detectors to capture monochromatic scenes, as in the case of fluorescence tests, increasing the cost of the equipment. Despite the sharpness of the retinographs and the high resolution of the images, retinographs are not present in most public places. They are intended for primary eye care, partly due to the purchase price, and many models have limited portability.

Digital photographic cameras that make up highly complex optical equipment for ocular image acquisition are also included in retinographs; these can capture detailed images of the eye in high definition, transferring the images to a computer and then printing them out, along with a report [67,68]. In companies that have the technology to manufacture these devices, there is a significant investment in research and development to increase the spatial resolution of these instruments, a factor mainly related to the quality of optical components and the capacity for high spatial resolution. In general, the exam is performed with the patient’s eye under mydriasis, with the iris dilated under drug action and the accommodation of the lens paralyzed. Image capture without the administration of drops is induced by keeping the patient in a dark environment, since the illumination of the retinograph is in an infrared region where the eye has low sensitivity, the iris has minor sensitivity, and its diameter is slightly reduced. However, after the first photo, the iris is sensitized.

The recent advances in access to telecommunications have resulted in an exponential increase in cheaper technologies, with better portability and technological improvement of background photographs, allowing increased access and the ability to track various ocular pathologies in more distant locations.

### 3.2. Optical Coherence Tomography (OCT)

Optical Coherence Tomography (OCT) is a diagnostic test used in several retinal pathologies [69]. For example, in glaucoma, OCT is used in suspected cases with structural alteration proven by fundus photography (retinography) when the cup/disc ratio is ≥0.5 and <0.9, in the presence of asymmetry between the two eyes of ≥0.2, localized thinning of the neural ring, or for diagnostic clarification in ocular hypertensive patients (ocular pressure above 21 mmHg).

The OCT makes it possible to diagnose and monitor glaucoma through monitoring the evolution of macular thickness [70]. Although central vision is often preserved in late glaucoma, thinning of the macular area is a parameter found in the early stages of the disease, and it precedes visual field defects.

### 3.3. Heidelberg Retinal Tomography (HRT)

The Heidelberg Retinal Tomography (HRT) is another diagnostic method used for the precise observation of the optic nerve head (especially for glaucoma). The HRT takes 3D photographs using a laser to document the optic nerve and the nearby retina. Using images of the desired deep layers, the instrument reconstructs a 3D image of the optic nerve. The HRT can be used to measure the area of the optic disc, cup, and the nearby area of the rim around the cup. The changes in the area can be computed after several visits from the patient [71].

### 3.4. Lenses

Lenses are relatively inexpensive optical elements that allow the visualization of some structures of the eye, but their individual use requires other optical systems to achieve the desired objective [72]. For example, 20 diopter ocular lenses can record sharp images of about 20 degrees from the posterior pole in the eye with a dilated pupil when kept at a certain distance from the patient’s eye, which causes some difficulty in the handling of the cell device. In addition, the 75 and 90 diopter ocular lenses allow 3D visualization of the optical papilla, but require a slit lamp.

### 3.5. Slit Lamp

The slit lamp is an optical system for studying anterior structures of the eye, such as the conjunctiva, cornea, anterior chamber, lens, iris, and posterior chamber as the vitreous body through luminous sections with the regulation of the slit thickness and the change in the angle of light incidence [73], allowing for the study of the retina and optical papilla when used with 75 and 90 diopter lenses (background biomicroscopy). The camera attached to the lenses will enable the capture of 2D and 3D images.

### 3.6. Direct Ophthalmoscope

The direct ophthalmoscope (average price from USD $200 to USD $300) has an opening through which objects on the other side can be seen (observer window) and a light source at the same point of the opening that allows illuminated viewing of the fundus of the eye [74]. The aperture is equipped with a set of lenses for refractive correction (focus control). Lateralization of the device in the optical axis of the patient’s eye allows the visualization of the peripapillary region. Under miosis (non-dilated pupil), the direct ophthalmoscope allows for a five-degree view of the retinal imaging field.

### 3.7. Binocular Indirect Ophthalmoscope

The binocular indirect ophthalmoscope (average price from USD $1000 to USD $3000) has a structure to attach to the examiner’s head, two openings, through which can be seen what is on the other side (observer window), and a light source above the openings that illuminates the observer’s field of view and the patient’s fundus, with the pupil dilated, using a 20 diopter lens, and providing a 3D image [75,76].

Background biomicroscopy (average prices: slit lamp USD $3000 to UDS $5000 and lenses USD $300 to USD $600) uses 75 and 90 diopter lenses to allow observation of the papilla in 3D, even with the pupil not dilated [77].

Table 2 lists the diagnostic equipment with the respective main characteristics.

### 3.8. Mobile Devices

More recently, equipment and lens systems have emerged that can be attached to smartphone cameras; these are light and easy to handle and transport, enabling images to be stored and transmitted to specialists via the Internet. On current smartphones, the success of papillary photographs may be limited by eyes with small pupils or by the closing movements of the pupil in the light from the device’s flash.

Because they are cheaper and more portable, smartphones are routinely used in ophthalmology services, and their portability and easy handling features allow for obtaining ocular images. With the advancement of technology, the smartphone camera has become a helpful tool in the ophthalmological office. It is lightweight, portable, easy to handle, and allows for control of photographic parameters. The images obtained can be stored on the cell phone or transmitted via the Internet to other systems and other specialists in more distant locations. It is necessary to attach a special lens to eliminate distortions and condense the image on the smartphone’s optics to take the picture. The photographs obtained are 2D.

Smartphones can serve as the direct ophthalmoscope when attached to a lens in optical systems. Examples of lenses attached to smartphones are the Welch Allyn iExaminer [78], the Ocular Cellscope [79], the Portable Eye Examination Kit [80], and the D-Eye System [81]. Regarding the Ocular Cellscope, the optical portion of a cell phone is integrated with a camera. The D-Eye System’s D-Eye adapter connected to the smartphone can take pictures with an approximate field of 20°, with the pupil dilated. When the D-Eye lens is attached to a cell phone, 2D images of the optic papilla can be obtained, even with the pupil not dilated, allowing for the screening of the glaucomatous papilla. Among the limitations of the use of cameras coupled to smartphones are the difficulty in aligning the image with the device and the strong flash intensity of the smartphone, which causes pupillary miosis at the time of photography. In background biomicroscopy, the smartphone objective coupled with the slit lamp objective allows the photographing of the patient’s optical papilla, observed through 75 and 90 diopter lenses, and in indirect ophthalmoscopy, the structures of the pole can be photographed as viewed through a 20 diopter lens.

Low-resolution retinal images obtained using low-cost, light, and portable devices can offer advantages in terms of diagnostic accuracy and increase the number of people served [82,83]. However, images produced through miotic pupils (without dilation) bring greater comfort to the patient, but lower quality because of ocular means, opacities, and difficulty in patient collaboration. In addition, although many studies have already been published, more robust evidence is still lacking about the reproducibility of automatic methods and the difficulty in segmenting the optic papilla and retinal vessels in low-resolution images, due to anatomical variability.

Table 3 presents some portable devices that allow the acquisition, storage, and transmission of retinal images.

### 3.9. Other Equipment

Fundus images without pupillary dilation (non-mydriatic cameras) can photograph the papilla and peripapillary region without the need to artificially dilate the patient’s pupil because of the size of the pupillary aperture [90].

Artificial pupil dilation with mydriatic drops allows for visualizing a wider retina and posterior pole field [91]. In screening for glaucoma, the observation of the papilla is considered the gold standard used in the diagnostic routine for the recognition of glaucomatous characteristics by specialists. However, there are more expensive and sensitive methods for detecting fiber layer damage, such as optical coherence tomography (OCT) [92], red-free retinal nerve fiber layer (RNFL) photography [93], stereoscopic photographs [94], or visual field tests.

Ophthalmoscopy allows the proper visualization of a more restricted field of the retina [95], including the optic papilla, even without pupil dilation. The devices converge and eliminate distortions in the patient’s retinal images and are captured by the observer’s eye or by a camera. The direct ophthalmoscope allows for the viewing of a magnified 2D image of the optic papilla and a reduced field in transparent media. In background biomicroscopy and indirect ophthalmoscopy, the observer has a stereoscopic and smaller view of the papilla. The greater the lens’ dioptric power, the more detailed the image of the structure in the photograph will be, but the field of view will be more restricted.

The observation of the posterior pole and the optical papilla by an external observer is carried out through optical systems that transform the refractive aberrations of the site to be observed in the eye into clear images for the examiner, also enabling the acquisition, storage, and sending of images to more places [96]. The observation of the optic papilla can be performed with the pupil undilated, or dilated using mydriatic drops. The pupillary slit controls the entry of light into the eye. Usually, the pupil contracts when exposed to an intensity of light that stimulates the direct pupillary reflex, as when the pupil is exposed to “flashes” from photographic equipment; therefor, these devices require adjustment for a slower action of the photographic system diaphragm to avoid the image being obtained with the pupil contracted. The pupillary slit can vary in diameter between the eyes in an environment with the same light intensity. The pupil can be artificially dilated through mydriatic drugs used during the eye exam to treat inflammatory processes of the anterior segment, iritis, iridocyclitis, or uveitis to allow for intraocular lenses. In photography, the dilated pupil is used to obtain a larger field in retinal and lens images. There are cheaper and more portable optical resources that can be used to analyze visual papilla images, such as the direct ophthalmoscope, background biomicroscopy with 75 and 90 diopter lenses used in a slit lamp, and the indirect ophthalmoscope using a lens of 20 diopters. However, they still require manual fixation to maintain the focal distance of the object, with prejudice to the handling of the portable optical device.

The most expensive optical resources include the retinographs containing a camera that photographs the fundus of the eye, with or without dilated pupils, using high-magnification lenses that capture 3D images of the retina with superior quality as compared to the devices already described. Some models incorporate digital features to capture, treat, store, process, and print retinal images and include autofocus and auto-capture features. Non-mydriatic retinographs can reach fields of about 20 to 40 degrees, and even larger fields under mydriasis. They offer portability, but they are expensive devices, and they are not available in many public primary ophthalmology services.

The technological evolution of photographic cameras allows for taking photographs of the papilla through portable cameras:

- **Advantages:** with low cost and easy handling, they can be incorporated into smartphone cameras, with low price, good quality, and the capability of transmission to other specialists, yet without diagnostic disadvantages regarding stereoscopic photographs.
- **Disadvantages:** However, taking photographs using portable cameras requires some extra technical training from a professional for the correct alignment of ocular structures.

The authors of [97,98] concluded that fundus images obtained by smartphones have strong agreement with the gold standards for clinical or photographic examination, which can expand patients’ access to medical care through photographs taken by smartphones, favoring the service of populations farther away from large centers through the transmission of images (telemedicine).

Both can help to obtain 2D photographs of the papilla. Portability favors the use of this technology in more distant populations. The use of cell phone technology allows for pre-adjustment, depending on the size of the papilla, the level of the refractive condition (ametropia), flash intensity, storage, and transmission of the image over the Internet to other centers. However, the disadvantages of this technology include requisite prior training, limited papillary imaging for small pupils, difficulty maintaining focus during involuntary eye movements, lack of patient cooperation, and ineffectiveness for pathologies exhibiting media opacity.

## 4. Current Studies for Automatic Tracking of Glaucoma

When glaucomatous neuropathy progresses, the cupping region (inner component) enlarges in comparison to the optic disc’s expansion (outer component). The neural rhyme is the space between the cup and the papilla’s outer edge. It is constituted of around 1,200,000 ganglion cell fibers from the retina that extend via the optic nerve, conveying visual information to the occipital lobe of the brain, where it is then interpreted.

Below are the most used indicators for glaucomatous papillae evaluation:

1. Cup-to-Disc Ratio (CDR)—a ratio between the vertical or horizontal diameter of cupping and the papilla that can indicate the existence of glaucoma [19,31,58,81,99,100].
2. Cup-to-Disc Area Ratio (CDAR)—follows the same premises of CDR comparing the area of the two components instead of the diameter and indicating the existence of glaucoma [19,31,58,81,99,100].
3. Inferior, Superior, Nasal, and Temporal rule (ISNT)—characterizes the healthiness of the optic disc based on the thickness in certain regions (inferior, superior, nasal, and temporal poles). It can be an early symptom of disease if this pattern is disrupted (in this order), either by a change in diameter or area [42,56,101].
4. Disc Damage Likelihood Scale (DDLS)—the risk of optic disc injury is calculated by comparing the diameter of the neural rim and the optic disc and the shortest distance between the optical disc contour and the excavation [43,102].

In the case of the use of CDR, the increase in the ratio between the optic disc and the cup is an indicator of disease risk, and it is sensitive to assessing progressive neural losses that occur in glaucoma [99]. However, the ability to detect glaucoma through CDR is limited due to variability in the analysis between the examiners (which could reach 0.2 or more) and by the fact that high CDR value in normal eyes can be explained by the correlation of the size of the optic disc and the age group. Eyes with large optic discs have a larger CDR, tending to have larger dimensions than eyes with smaller discs. Even in normal eyes, there is loss of neural tissue due to the age at which it is replaced by scar tissue, indistinguishable from neuronal tissues on clinical examination, suggesting that CDR evaluation should be used along with other glaucoma detection methods such as intraocular pressure, drainage angle, presence of optic nerve damage, peripheral loss of vision (visual field test), and computerized imaging of the optic nerve and corneal thickness [100].

Ophthalmologists use methods that can assist in the evaluation of the papilla based on characteristics determined by color, texture, optic disc, and cup delimitation. First, these metrics are calculated through direct observation of the papilla and a comparison of the two eyes of the patient. The delimitation of the exaction region compared to the optic disc can be more difficult, since the subtlety of the nerve fiber layer causes imprecision in the cupping limits.

However, segmentation methodologies (manual or automated) have some restrictions, namely, low resolution, opacity, and photographic artefacts (e.g., imaging lighting and distortion) that can influence the segmentation precision. Blood vessels and variation in the intensity of the delimitation edge color make cupping segmentation a challenging task. Bock et al. [103], inspired by facial and object recognition, achieved 75% sensitivity and 85% reproducibility in detecting glaucomatous papillae without requiring papilla segmentation, depending on more sophisticated equipment. Since the papilla is more susceptible to errors due to the difficulties mentioned previously, many positive and negative glaucoma samples are required for screening, making the recognition of the papilla based on appearance challenging. In the future, this can be solved by using more advanced technology to acquire high-resolution images, allowing for glaucoma to be identified simply by the appearance of the fundus image.

Automatic segmentation methods for the measurement of the optic disc and cupping can track, identify, and evaluate the evolution of glaucoma, despite this being a complex process due to the subtlety of variations in the anatomy of these components, namely the nerve fiber layer, the notch, and the presence of blood vessels that can in some cases, cause the delimitation of the cupping area, and in other cases, can help to visualize the limits of the excavation by the deflection of the atrophy of the nerve fiber layer. Image processing for the optic disc segmentation can include the extraction of blood vessels, normalization of the lighting, contrast enhancement, and selection of an image channel.

Claro et al. [104] divided segmentation techniques into five groups: the superpixel methods (including studies for segmenting the optic disc and cupping regions), followed by clustering, mathematical morphology, active contour, and convolutional neural network methods, without a consensus on the best approach.

Many of these techniques have some restrictions due to the sharpness variation for the optic disc and adjacent region boundaries, inconstancy in the optic papilla structure in normal eyes, the attendance of peripapillary atrophy, the papillary drusen (structures limiting the optic disc) which may cover the optic disc completely in advanced cases, and the blood vessel paths (whose deflection play a key role for excavation boundaries), all of which can make the delimitation of the papilla inner border difficult. Either way, these retinal pathological changes (diffuse papillary atrophy, peripapillary atrophy, papilla insertion changes, and papilla drusen), especially near the optic nerve, should be considered to obtain correct CDR and ISNT measurements for glaucoma screening

According to Almazroa et al. [49], the major limitation of the current methods is the limited number of datasets (DRIVE and STARE) that do not provide images exhibiting many different characteristics. The segmentation process is even more complex due to the low resolution of the images (from 0.4 to 0.3 megapixels). Most of these images were acquired from adults, and in most cases, without images of both eyes from the same patient. Retinal images from children and babies have different anatomic characteristics (compared to adults) that must be considered for segmentation techniques.

Table 4 summarizes the glaucoma indicators described within the respective papers, with an explanation of each one.

Machine learning algorithms can effectively deal with large multi-modal datasets, making it easy to solve problems that would be impractical through classical statistical analysis. As a subarea of machine learning, deep learning processes the data through multiple neural network layers. Such algorithms quickly became a standard methodology for analyzing medical images for image classification, object detection, segmentation, registration, and other tasks.

Deep learning can identify features within a complex structure in large datasets using multiple intermediate layers positioned between the input and output layers, allowing each level to learn to transform its input signal into the next layer.

A fundamental prerequisite for the successful application of these methods is the availability of a sufficient amount of data to train and test the system. Furthermore, an open challenge is the validation of these methods, which requires a reference standard that can be used for comparison [105].

Deep learning has demonstrated broad potential for applicability in the medical field by enhancing image accuracy, processing, and identification of elements of diagnostic relevance in radiographic images, tomography, ultrasound, histological analysis of organs and tissues, and the analysis of photographic images.

Notably, these imaging tasks are used in the following application areas: neuro, retinal, pulmonary, digital pathology, breast, cardiac, abdominal, and musculoskeletal imaging, among others [105].

An important property of medical data is its multimodality, because it can provide a wealth of information about a target (e.g., tumor, organ, or tissue). Segmentation is a common task in deep learning based on medical data, and sometimes, it is a step in a more complex data processing pipeline. Using multimodality consists of fusing multi-information to improve the segmentation [106].

## 5. Challenges for Glaucoma Screening

The best method for obtaining images in mass tracking of the glaucomatous papilla would be a system that allows for the obtaining of high-quality photos without the need for pupil, in a manner that is easy to handle and transport, inexpensive, reliable in detecting early/moderate forms of glaucoma, fast, and which provides reasonable accuracy.

### 5.1. Lacking Data, Need to Standardize

The existence of different configurations between the datasets of the databases and the lack of data that point out physiological differences within normal patterns and pathological findings can represent bottlenecks that distance the databases from real-life conditions. Technological advances, including cameras with better photo quality that also offer portability, less aberration of images, and an expansion of the optical field, even without pupil dilation, may be inclusive factors for analysis, even by non-specialists. Even though these methods can be used by ordinary people, the results still need more robust multicentric studies, with analysis of papillary images obtained from different ethnic groups and groups of various specialists. Further analysis of the automated methods used for correlating different algorithms for better data correlation is also required, as well as the inclusion of clinical aspects in the process of learning, with an increase in individual data concerning race, family data, personal complaints, and data from anamnesis that will allow for the diagnosis of glaucomatous disease, particularly in the earlier forms [107].

### 5.2. Integration of Historical and Anamnesis Data

Another challenge to be clarified is how ophthalmic history and anamnesis data can be incorporated into the automated analysis to bring more reliable, practical results in the study of glaucoma progression [108,109] in the early stages, where changes in the nerve fiber layer and changes in the excavation of the papilla are barely visible, making diagnosis difficult by studying glaucomatous changes through the nerve fiber layer.

Since medical science is evidence-based, glaucoma screening requires more physiological and pathological data to add to the dataset to make automated analysis feasible.

### 5.3. Development of Methods “That Learn Continuously”

More reliable results can be obtained when clinical image data such as angiography, optical coherence, visual field, and background images are integrated to build an artificial intelligence system for a generalized and more reliable diagnosis. In the age of evidence-based medicine, it is difficult for doctors and patients to trust a methodology that cannot explain the diagnosis. Therefore, must be built AI platforms using varied multimodal data to approximate the real clinic [107].

### 5.4. Development of “Explainable AI” Methods

Current medical knowledge derived from decades of observational data collection, hypotheses, and validation through clinical research cannot simply take the findings of a deep learning system as absolute in accuracy without understanding, evaluating, and validating the results to be consistent with prior knowledge. Therefore, deep learning solutions cannot replace, but should complement the doctor-patient relationship based on trust and compassion [107].

### 5.5. Development of Methods to Detect Multiple Diseases

While the accuracy of the results is promising, it is necessary to remain prudent and sober when deploying these models in the real world, where numerous forms of retinal disease and glaucoma exist. Studies based on the intelligent diagnosis of eye diseases using artificial intelligence systems need to be constructed to detect different retinal diseases with high precision. In addition, new methods using varying qualities of imaging must be urgently developed.

## 6. Conclusions

Several factors are highly correlated with the presence of glaucoma, namely, age, family, medication, and ocular factors. Deep learning solutions are mostly based on the optic papilla appearance, without considering the above-mentioned risk factors.

In fact, the visual characteristics of the papilla are an extremely significant factor for glaucoma screening, especially in more intermediate and advanced stages, but risk factors must be considered, just as they are in clinical practice. However, some other researchers presented interesting results by combining visual characteristics of glaucoma papilla and data from anamnesis, coming closer to a real diagnosis in the clinical environment.

When the advantages outweigh the disadvantages, the treatment is justified, proving that AI can help in the screening for glaucoma and the monitoring of its treatment, based on the previously mentioned characteristics, even though other parameters may be required. When the AI system or the ophthalmologist find characteristics of glaucoma, the treatment must be started in order to prevent the aggravation of the disease. The decision can be more delicate in doubtful cases, leading to unnecessary treatment, even though the risks point to the presence of glaucoma, and in these cases, the AI system can provide more significant assistance in the screening process.

In the future, more contributions regarding deep learning to assist in glaucoma screening will be developed, since more data has been acquired, hopefully integrating different modalities to enhance the confidence level in these systems. It is important to include both retinal images and known risk factors, as well as different image modalities from another types of equipment, to improve these AI screening systems in order to increase the aptitude of glaucoma screening.

## Figures and Tables

**Table 1:** Example of anamnesis data.

Type	Collected Data
External eye inspection	eye movements, opacities, and eye volume change
Eye complaints	visual blurring, presence of colored halos, vision loss
Personal background	existence of chronic diseases (e.g., diabetes, hypertension, neurological and rheumatologic conditions) and use of medications (e.g., steroids, which increase the incidence of glaucoma)
Family background	incidence of glaucoma in first-degree relatives
Eye exam	eyeglasses, funduscopy with direct visualization of the optical papilla; biomicroscopy performed with the aid of a slit lamp that cuts the light at different angles and allows the verification of ocular structures

**Table 2:** Summary of equipment advantages, disadvantages, costs, and portability.

Equipment	Advantages	Disadvantages	Costs	Portability
Retinographer	Single image capture Excellent contrast and detail	Pupillary dilation may be needed Expensive Image quality more susceptible to media opacities, motion artefact, and image processing Cannot quantify membrane thickness and presence of edema	USD $50–USD $100K	No
OCT	Monitors evolution of macular thickness Improved resolution More images taken Eye tracking feature Portable	Expensive Limited penetration power Transverse resolution must to be similar to axial resolution	USD $10K–USD $50K	Yes
HRT	3D construction of optic nerve head Automatically tracks progression of the disease using contour lines sketched during previous patient examinations Real-time quality control during image acquisition Sophisticated analysis software for glaucoma detection and progression Large race-specific normative database that has been shown to improve diagnostics	Measurements rely on a reference plane based on the placement of a user-defined contour line Stereometric measurements can be influenced by moderate changes in IOP	>USD $5K	Yes
Lenses	Cheap Helps to increase angles of visualization	Requires other equipment for attachment	USD $3K–USD $5K	Yes
Slit Lamp	Easy examination of the eye structures in detail resolution improved in higher models by maximizing the quality of the lenses	Discomfort in some photophobic patients	>USD $5K	Yes
Direct Ophthalmoscope	High magnification Portable Check anterior and fundus Cheap	Pupillary dilation may be required Cannot use with ocular opacities Not quick	USD $0.2K–USD $0.3K	Yes
Binocular Indirect Ophthalmoscope	Greater area of the fundus Easier to use Ideal for defining the extent and height of retina High-quality stereoscopic image Portable Either magnification or field of view can be prioritised by varying the choice of the condensing lens	Not good for defining the relative depth of the lesion vertically and horizontally inverted image Complicates the recording of fundus abnormalities Level of magnification is relatively low High level of retinal illuminance can be uncomfortable for the patient	USD $1K–USD $3K	Yes

**Table 3:** Portable devices.

Equipment	Costs (k$)	Mydriatic	Field Angle	Lens (Diopter)	Resolution	Smartphone	CAD Software
Phelcom Eyer [84]	4	No	45°	N/D	12 MP	Samsung Galaxy S9	Integration with EyerCloud system
visoScope [85]	0.25/0.5	N/D	50°	20	N/D	iPhone	N/D
Volk Pictor Plus [86]	6.8	No	40°	N/D	2560 × 1920 pixels	No	N/D
Volk iNview [87]	1	N/D	50°	N/D	8 MP	iPhone 6 and 6S	N/D
Welch Allyn iExaminer [88]	N/D	No	25°	N/D	8 MP	iPhone 6 and 6S	N/D
D-EYE™ [89]	4	N/D	miosis 6° mydriasis 20°	N/D	8 MP	iPhone	N/D

**Table 4:** Summary of indicators of the presence of glaucoma.

Indicator	References	Nr of Papers	Explanation
CDR	[19,31,58,81,99,100]	6	Ratio between the vertical or horizontal diameter of cupping and the papilla.
CDAR	[19,31,58,81,99,100]	6	Ratio between the area of cupping and the papilla.
ISNT	[42,56,102]	3	Characterization of the healthiness of the optic disc based on the thickness in certain regions (inferior, superior, nasal, and temporal poles).
DDLS	[43,102]	2	Comparison of the diameter of the neural rim and the optic disc and the shortest distance between the optical disc contour and the excavation.

## Data Availability

Not applicable.
